# A Time to Pause and Reflect: When a Patient with Autoimmune Hepatitis Stops Responding to Corticosteroids

**DOI:** 10.1155/2016/7092434

**Published:** 2016-12-25

**Authors:** Lewis Tsang, Mitali Fadia, Shivakumar Chitturi

**Affiliations:** ^1^The Canberra Hospital, Garran, ACT, Australia; ^2^ACT Health Pathology, Garran, ACT, Australia

## Abstract

Drug-induced liver injury (DILI) with features of autoimmunity (AI) is a challenging diagnosis to make particularly due to its apparent corticosteroid responsiveness. We present the case of a 74-year-old woman who presented with a 2-week history of jaundice and fatigue. She was initially diagnosed with autoimmune hepatitis (AIH) based on biochemical and histological characteristics and prompt response with budesonide but a biochemical relapse occurred soon after inadvertent rechallenge with irbesartan, a drug that she had discontinued prior to her presentation but was not initially considered to be a cause of her symptoms.

## 1. Introduction

DILI-AI is an important type of liver disease. The main drugs that are known to cause this include minocycline, alpha methyldopa, nitrofurantoin, diclofenac, hydralazine, statins, and anti-TNF agents [1]. Distinguishing between a DILI-AI and AIH can be challenging particularly if the patient is on a variety of different agents. The mainstay of management is to identify and discontinue the offending drug.

## 2. Case Report

A previously well 74-year-old Caucasian woman presented with a 2-week history of jaundice and fatigue. She had no risk factors for viral hepatitis and did not drink alcohol or use herbal or dietary supplements. She had hypertension that was well controlled with irbesartan. Physical examination was significant for jaundice and hepatomegaly. There were no other peripheral signs of chronic liver disease. Laboratory tests at admission were as follows: normal full blood count, serum bilirubin 3.6 mg/dL, alanine aminotransferase (ALT) 1011 U/L, alkaline phosphatase 168 IU/L, albumin 37 g/L, globulin 51 g/L, IgG 31.1 g/L (normal, <16 g/L), and prothrombin time of 16 seconds. Antinuclear antibody titre was 1 : 5120 but smooth muscle, liver-kidney microsomal and mitochondrial antibodies were absent. Serologic tests for hepatitis A, B, and C were negative. A liver ultrasound showed normal sized bile ducts with no features of chronic liver disease.

**Figure 1:**
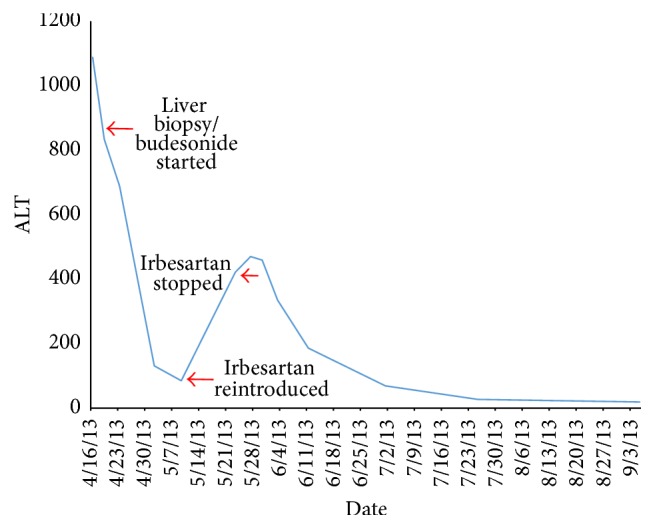
Timeline of ALT changes.

**Figure 2:**
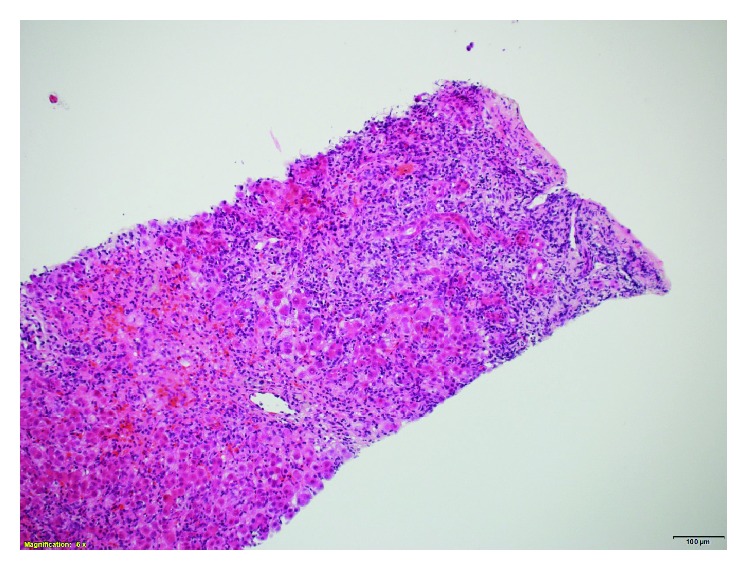
Liver biopsy showing severe interface hepatitis with moderate to severe mixed inflammatory infiltrates.

A liver biopsy on day 6 showed severe interface hepatitis with a mixed inflammatory infiltrates consistent with severe AIH (Figure 1). She scored 7 on the simplified diagnostic criteria for AIH which is consistent with “definite autoimmune hepatitis” [2]. Budesonide 9 mg daily was commenced. She began to improve both clinically and biochemically with her ALT dropping to 85 IU/L within 10 days. After this initial response, her ALT rose again to 421 U/L despite remaining budesonide (Figure 2). On closer questioning, it was later ascertained that irbesartan had been commenced 3 months earlier but had been discontinued 3 days before admission (9 days before her liver biopsy; ALT 834 IU/L at this time). After the initial response to budesonide, a diagnosis of AIH had been established and the irbesartan was recommenced, only to be followed by an ALT rise to 421 (within 2 weeks). A diagnosis of irbesartan-induced hepatitis was considered and the drug was withdrawn and her liver tests returned to normal within 6 weeks. Budesonide was tapered off over a period of 8 months. She remains well with normal liver tests 3 years after her initial presentation. The score for causality assessment for DILI using the updated Roussel Uclaf Causality Assessment Method (RUCAM) was 11 for irbesartan (highly probable) (Table 1) [3].

## 3. Discussion

Over 90% of patients with AIH will respond to immunosuppressive therapy. Therefore, any factors contributing to the loss of response need to be carefully considered. These include noncompliance with drugs, progression of the liver disease, and also other alternative diagnoses. While viral hepatitis can be readily excluded by serologic testing, a diagnosis of DILI still relies heavily on an accurate clinical history. The International autoimmune hepatitis group have developed criteria to aid clinicians in establishing a diagnosis of AIH.

This case illustrates the difficulties posed by applying such criteria to individual patients.

Although this patient fulfilled criteria for AIH and had a number of typical features of AIH including high-titre antinuclear antibodies, raised globulins, compatible liver histology, and an apparent response of steroids, the true nature of the liver injury became clear only after the initial response to steroids had begun to wane and the serum aminotransferases began to increase on rechallenge. Positive rechallenge is weighted heavily in causality assessment scales for DILI but is rarely justified due to its potential hazards [4]. However, as in this case, inadvertent rechallenge can still be valuable. The alternative would have been to place a 74-year-old patient on long-term immunosuppression. In the Mayo clinic series of 261 patients with AIH, 9% were cases of DILI-AI features [5]. In that study, corticosteroids could be ceased (without relapse) in all DILI cases (35% for AIH cases) and the authors considered this to be one of the distinguishing features between DILI and AIH.

This is nicely illustrated in this patient who has not needed corticosteroids after the initial course. The spectrum of DILI is broad and encompasses both acute and chronic liver disease. Patients presenting with DILI and autoimmune features represent a special subgroup of patients sharing many clinical, biochemical, immunologic, and histologic characteristics with other patients with AIH. Well-known drugs associated with AIH-like features include minocycline, alpha methyldopa, nitrofurantoin, diclofenac, hydralazine, and antitumor necrosis factor-alfa antagonists. However, there are other drugs that may occasionally present with DILI. This can lead to delays in recognition and alternative treatment strategies. The mainstay of management is timely identification and cessation of the offending agent. Irbesartan is an effective antihypertensive agent belonging to the category of angiotensin II receptor antagonists. Liver injury has been reported with irbesartan and other members of this group. The predominant pattern is hepatocellular but occasional instances of acute cholestatic hepatitis or cholestasis have been noted [6]. Most cases present within 1–4 weeks of commencing the drug. Fortunately, the liver injury usually resolves with drug cessation. Chronic liver disease has not been reported but prolonged cholestasis can occur.

## 4. Conclusion

Here we have described a case of liver injury following the use of irbesartan, a commonly used antihypertensive. While the histopathology was compatible with severe AIH and responded to corticosteroids, reintroduction of the agent caused a recurrence of liver injury. The patient then improved with cessation of irbesartan and was eventually weaned off corticosteroids with no relapses, therefore indicating a drug-induced aetiology.

There are some well-known medications which are associated with AIH but there are also other drugs which only occasionally will cause these symptoms, so it is important to consider DILI-AI as a possible differential in patients who exhibit signs of AIH if the clinical history involves newly commenced medications.

## Figures and Tables

**Table 1:** Updated RUCAM results for irbesartan.

Items for hepatocellular injury	Score
Time of onset from starting the drug: 5 to 90 days	2
Course of ALT after cessation: decrease ≥50% within 30 days	2
Risk factors	
No alcohol use	1
age > 55 yrs (74)	1
Concomitant drugs/ herbs: none	0
Search for alternative causes: HAV/HBV/HCV negative, hepatobiliary ultrasound normal, no history of alcoholism or recent acute hypotension	0
Previous hepatotoxicity of drug, reaction labelled in the product characteristics	2
Response to unintentional reexposure, doubling of ALT with drug alone	3
Total	11^*∗*^

^*∗*^A score of >9 suggests that irbesartan is highly probable as being the cause of DILI in this case.
